# Supplementary figures and images for: Inhibition of (interstitial) P2Y6 receptors attenuates fibrosis progression
Source: Pflugers Arch. 2026 Jun 17;478(7):57. doi: 10.1007/s00424-026-03187-8 (PMC13272249; doi:10.1007/s00424-026-03187-8)

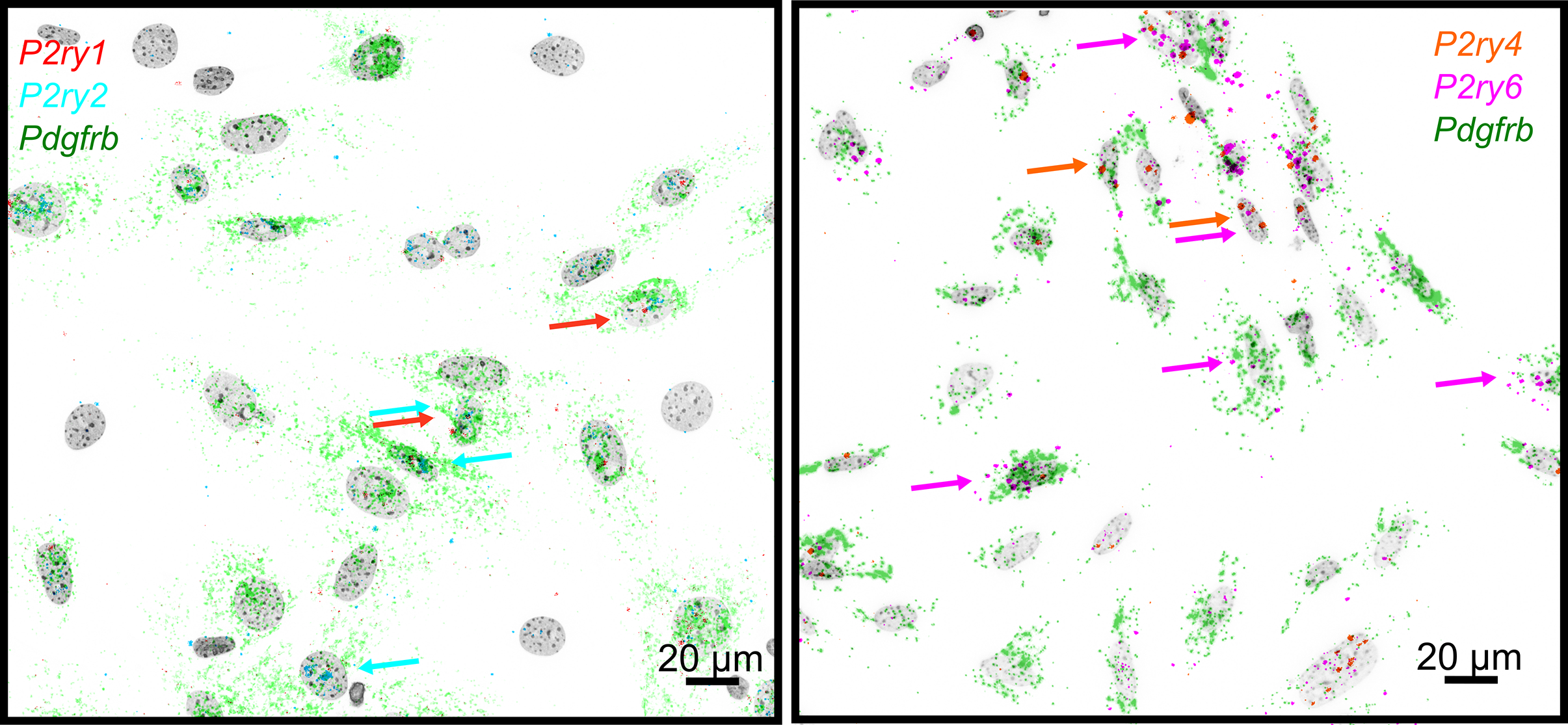

Supplement: Supplementary file 1 — (PNG 1.51 MB) [file 424_2026_3187_Fig6_ESM.png]

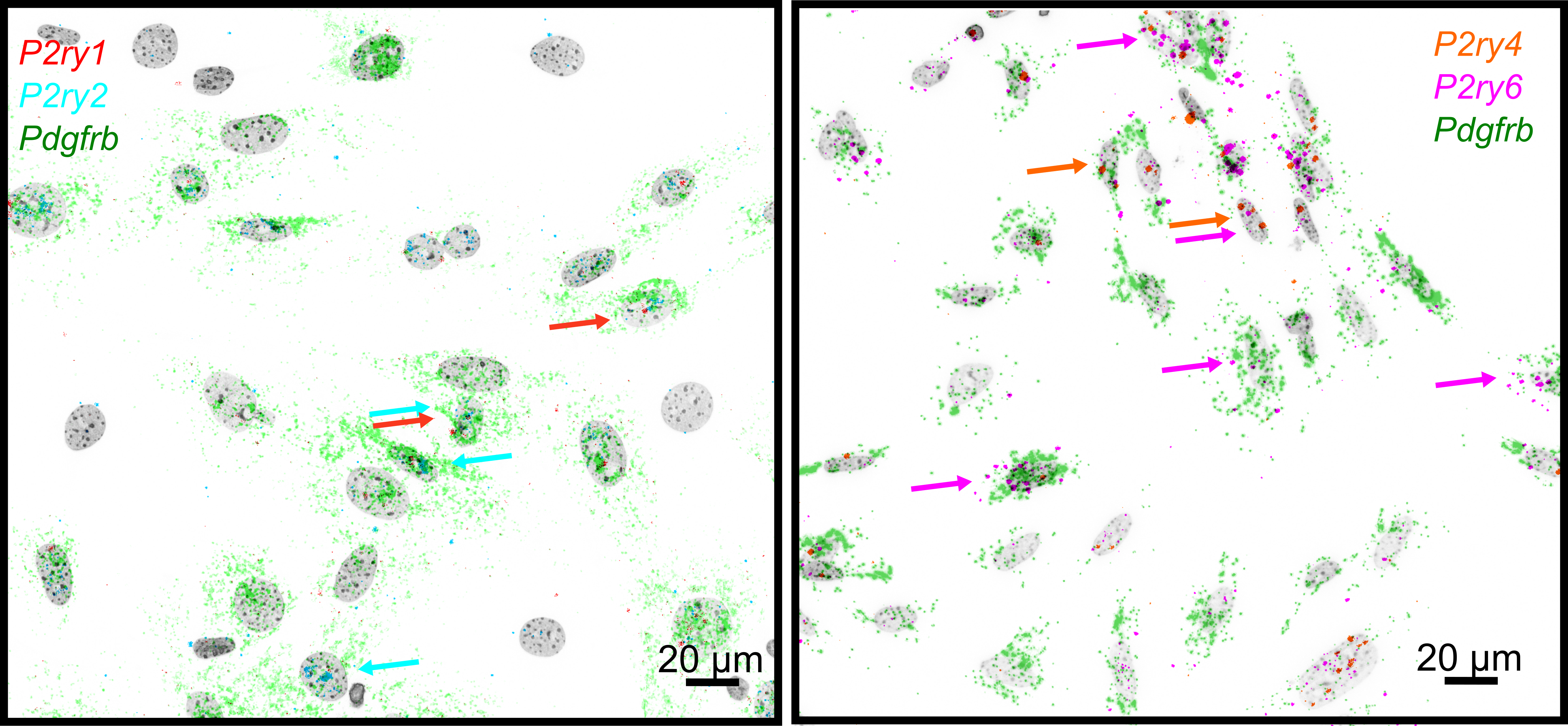

Supplement: Supplementary file 2 — Supplementary Material 1 [file 424_2026_3187_MOESM1_ESM.tiff]

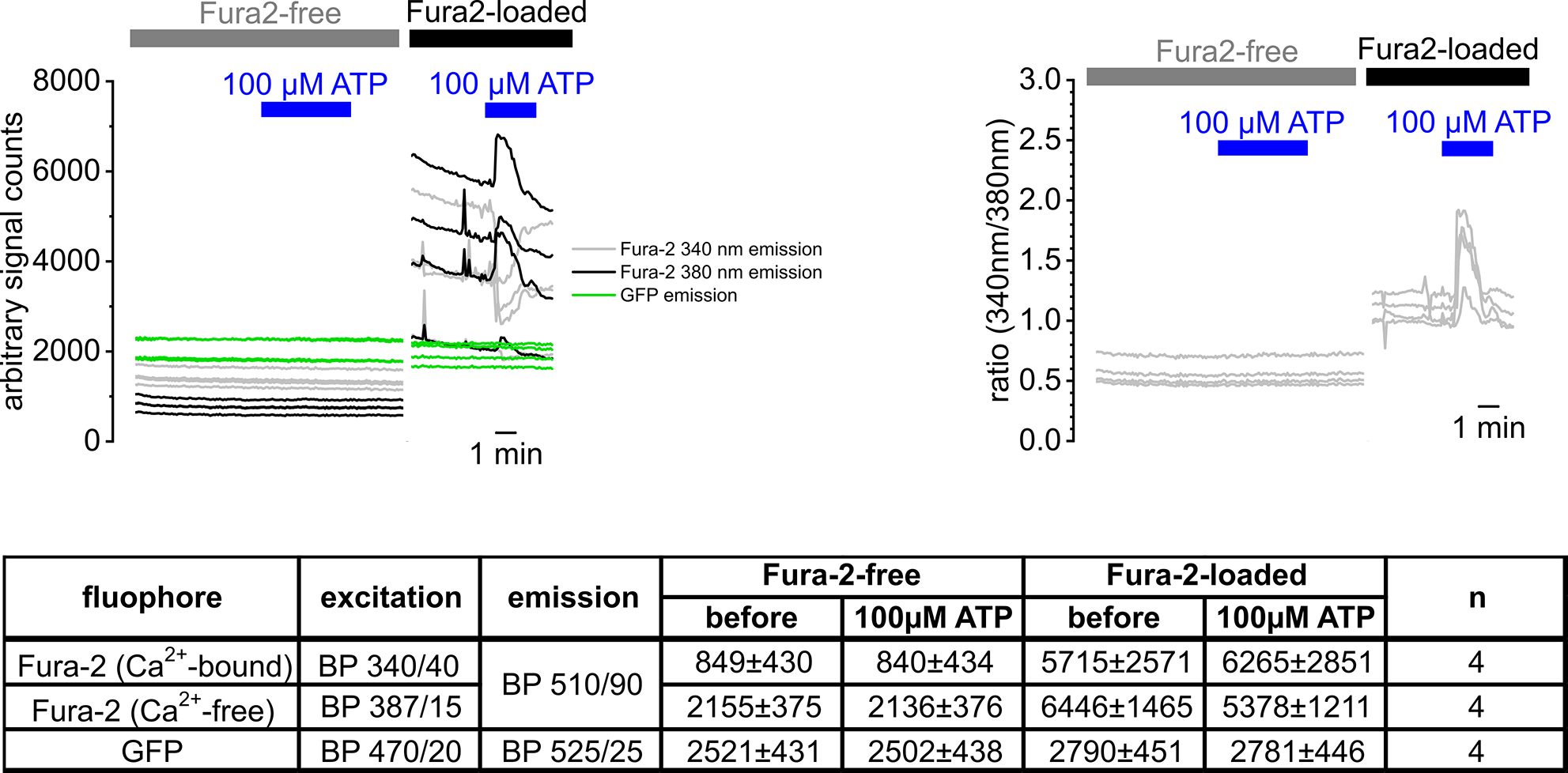

Supplement: Supplementary file 3 — (PNG 258 KB) [file 424_2026_3187_Fig7_ESM.png]

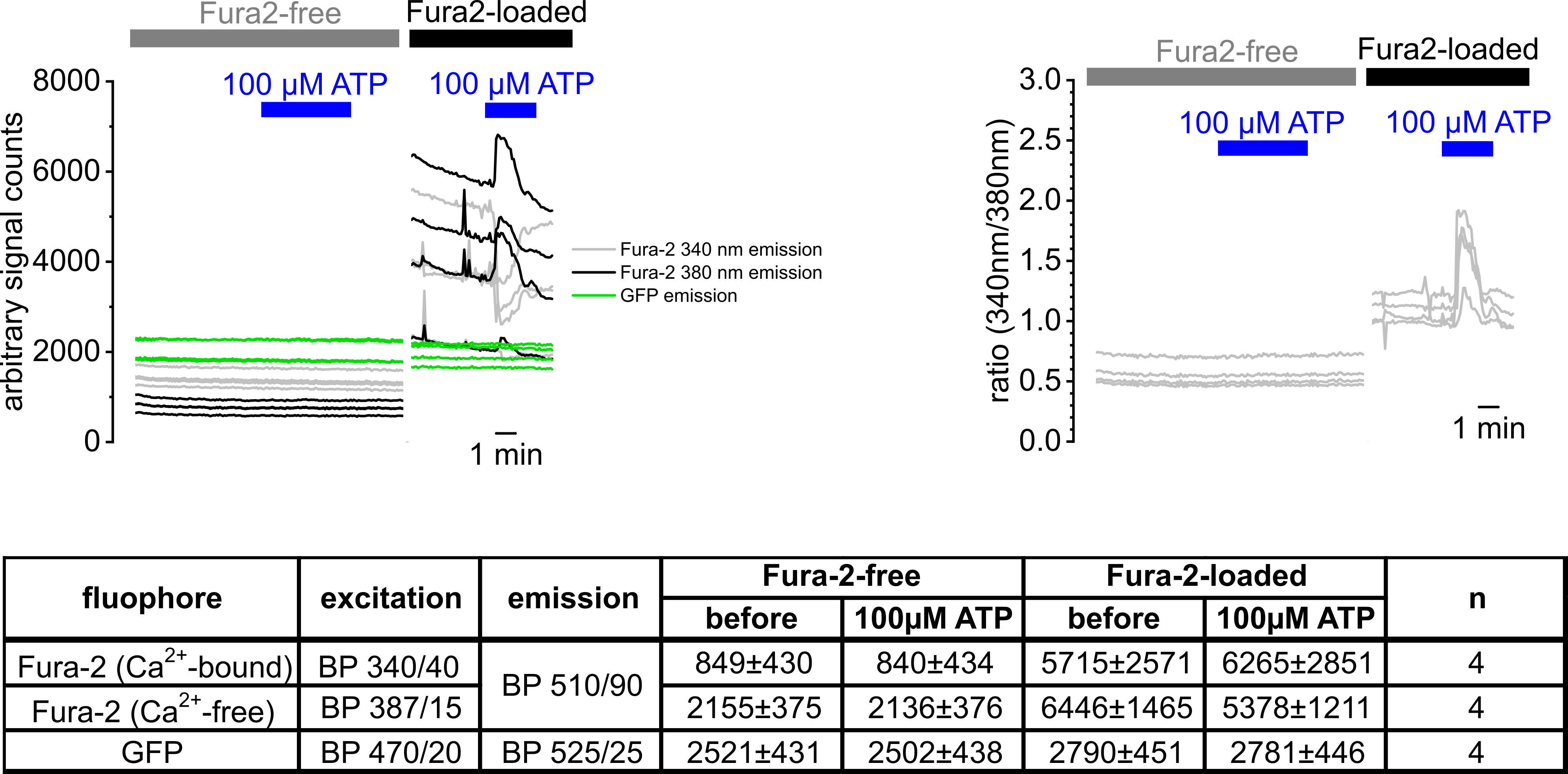

Supplement: Supplementary file 4 — Supplementary Material 2 [file 424_2026_3187_MOESM2_ESM.tiff]

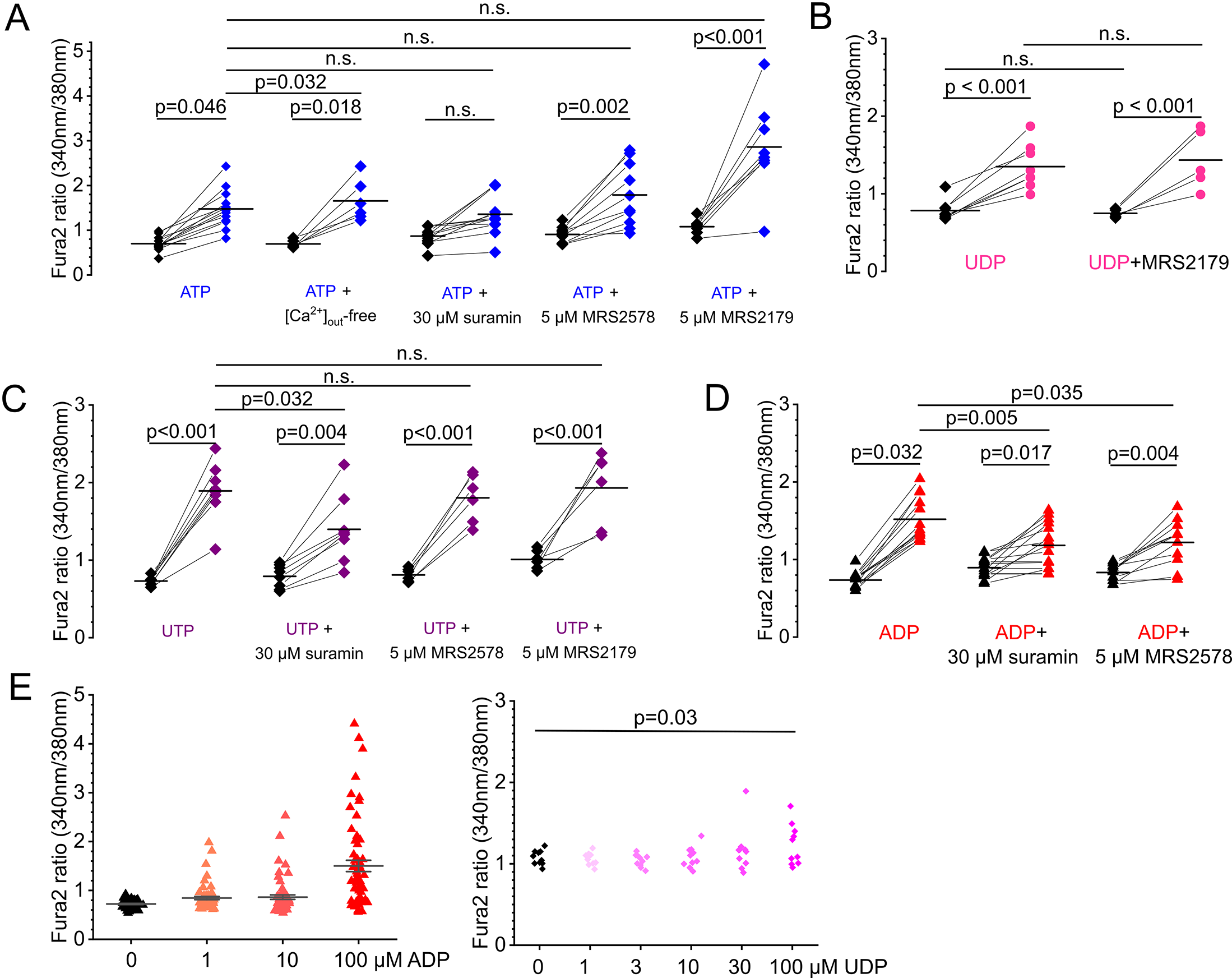

Supplement: Supplementary file 5 — (PNG 373) [file 424_2026_3187_Fig8_ESM.png]

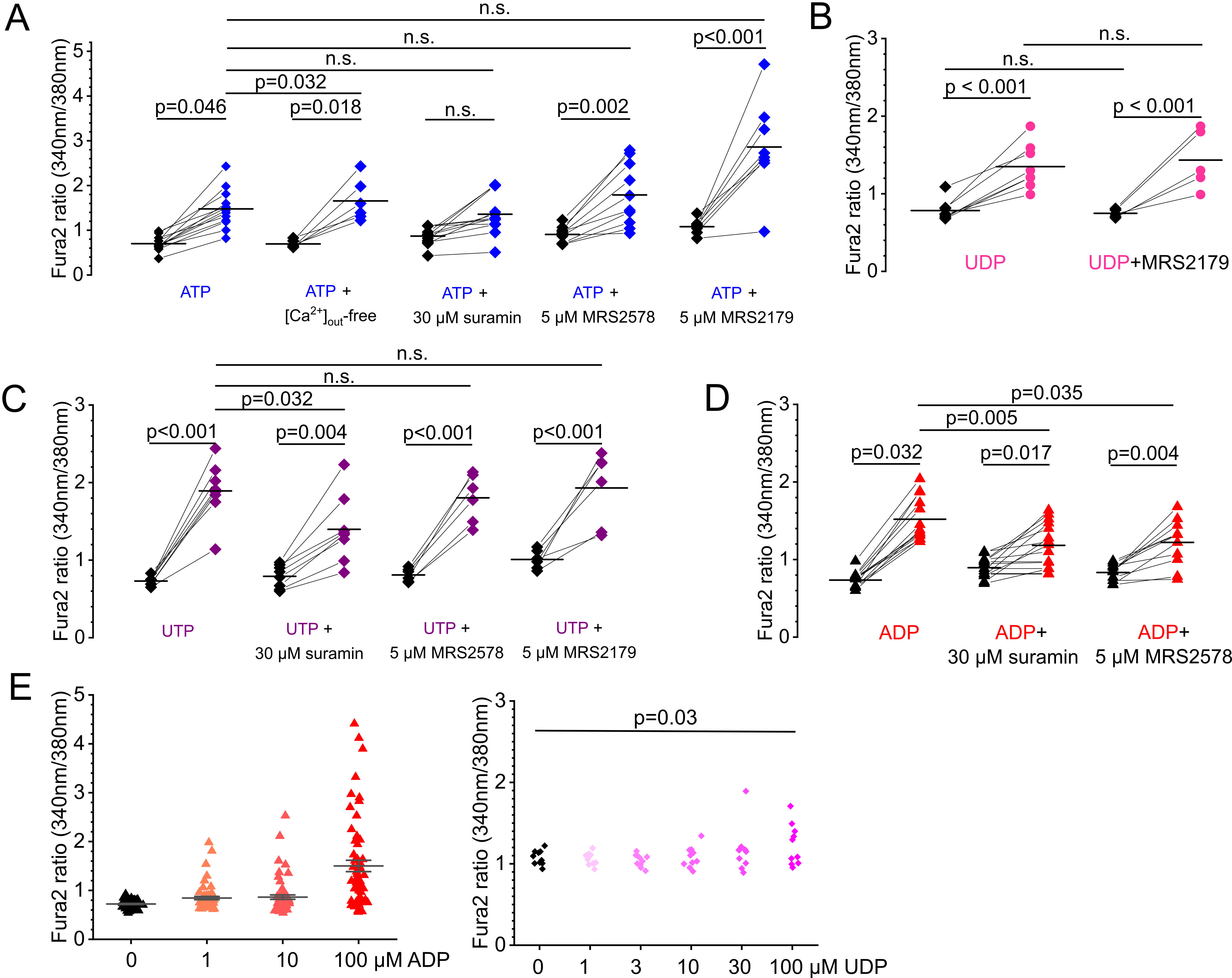

Supplement: Supplementary file 6 — Supplementary Material 3 [file 424_2026_3187_MOESM3_ESM.tiff]

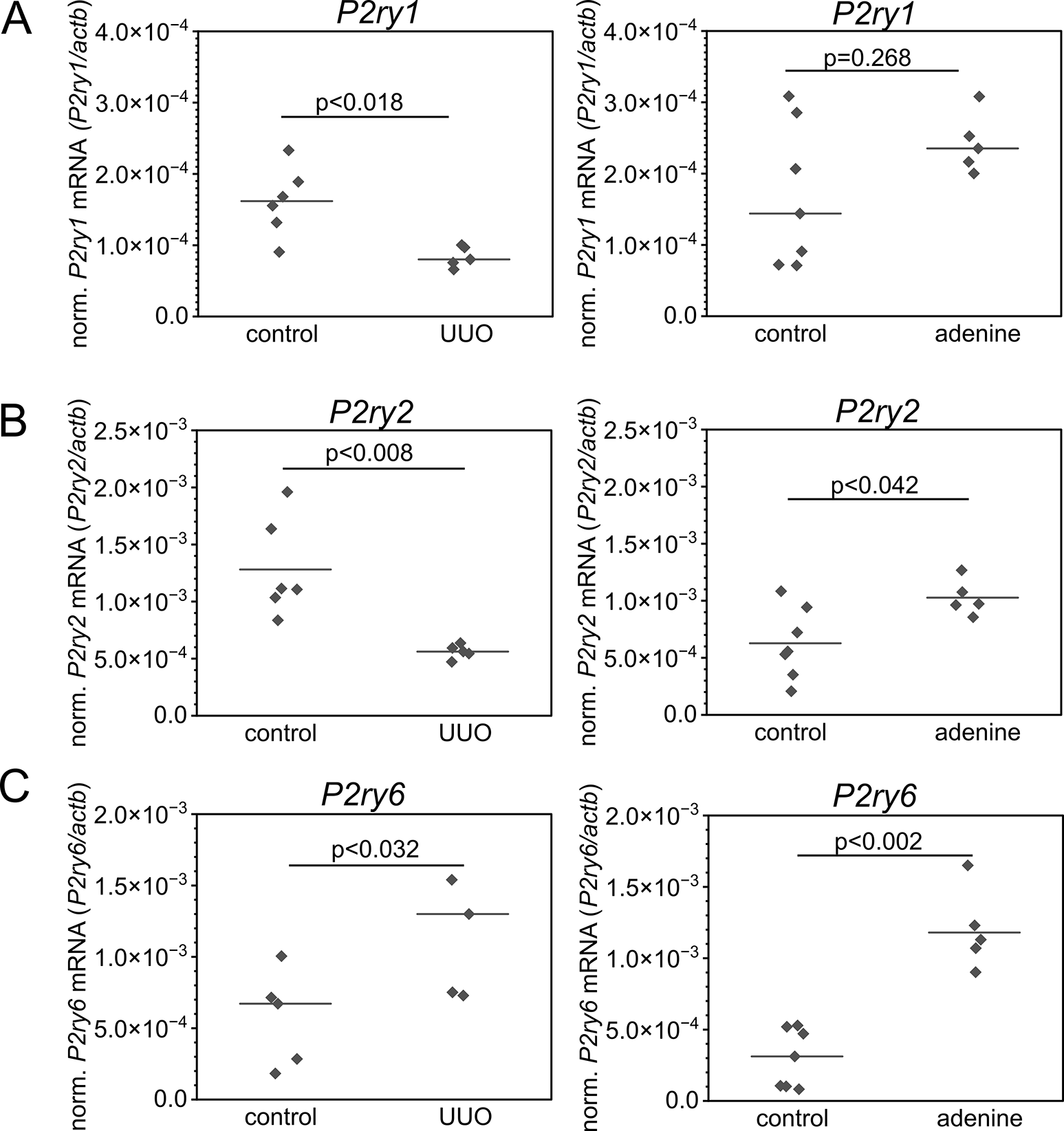

Supplement: Supplementary file 7 — (PNG 231 KB) [file 424_2026_3187_Fig9_ESM.png]

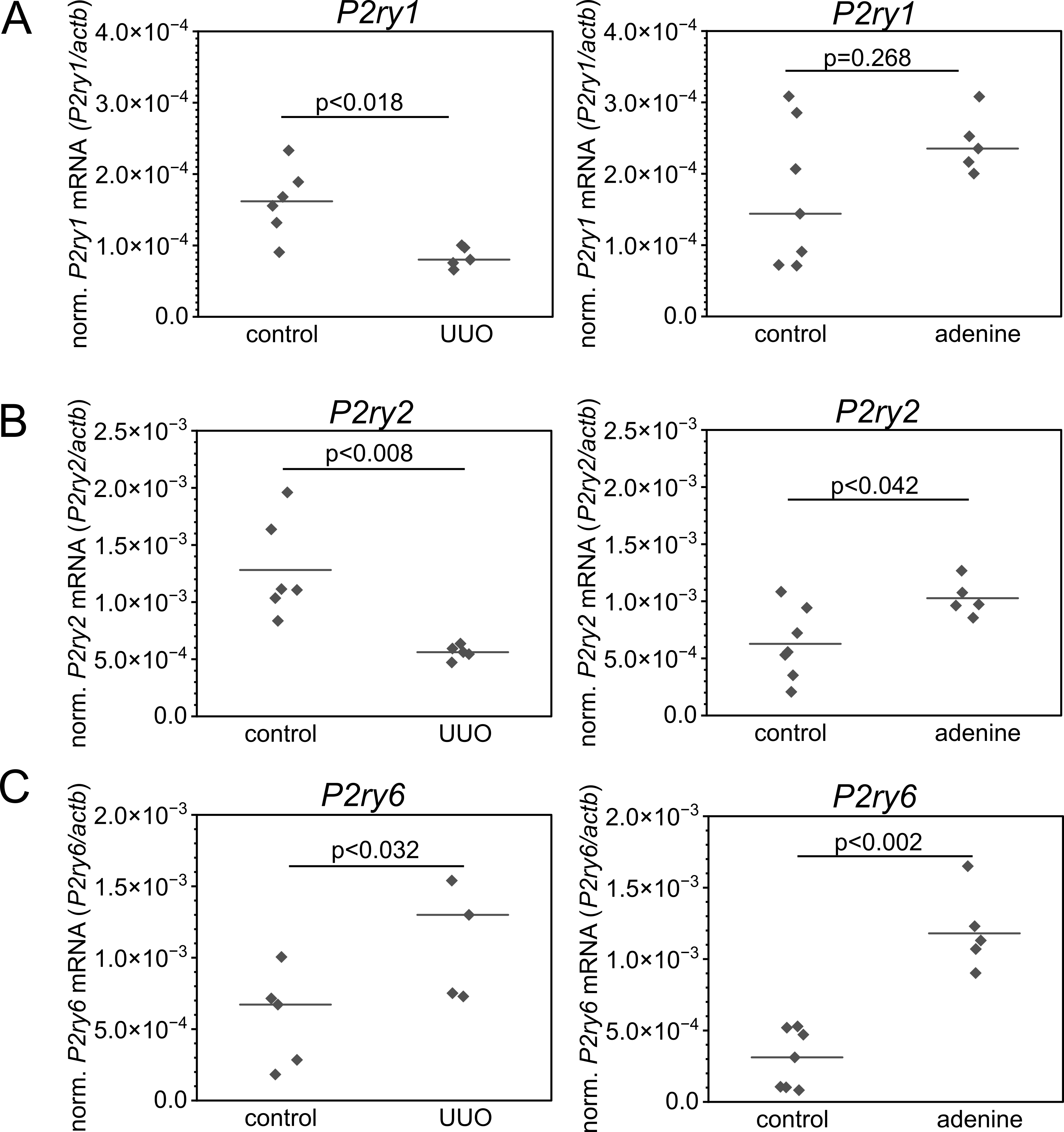

Supplement: Supplementary file 8 — Supplementary Material 4 [file 424_2026_3187_MOESM4_ESM.tiff]

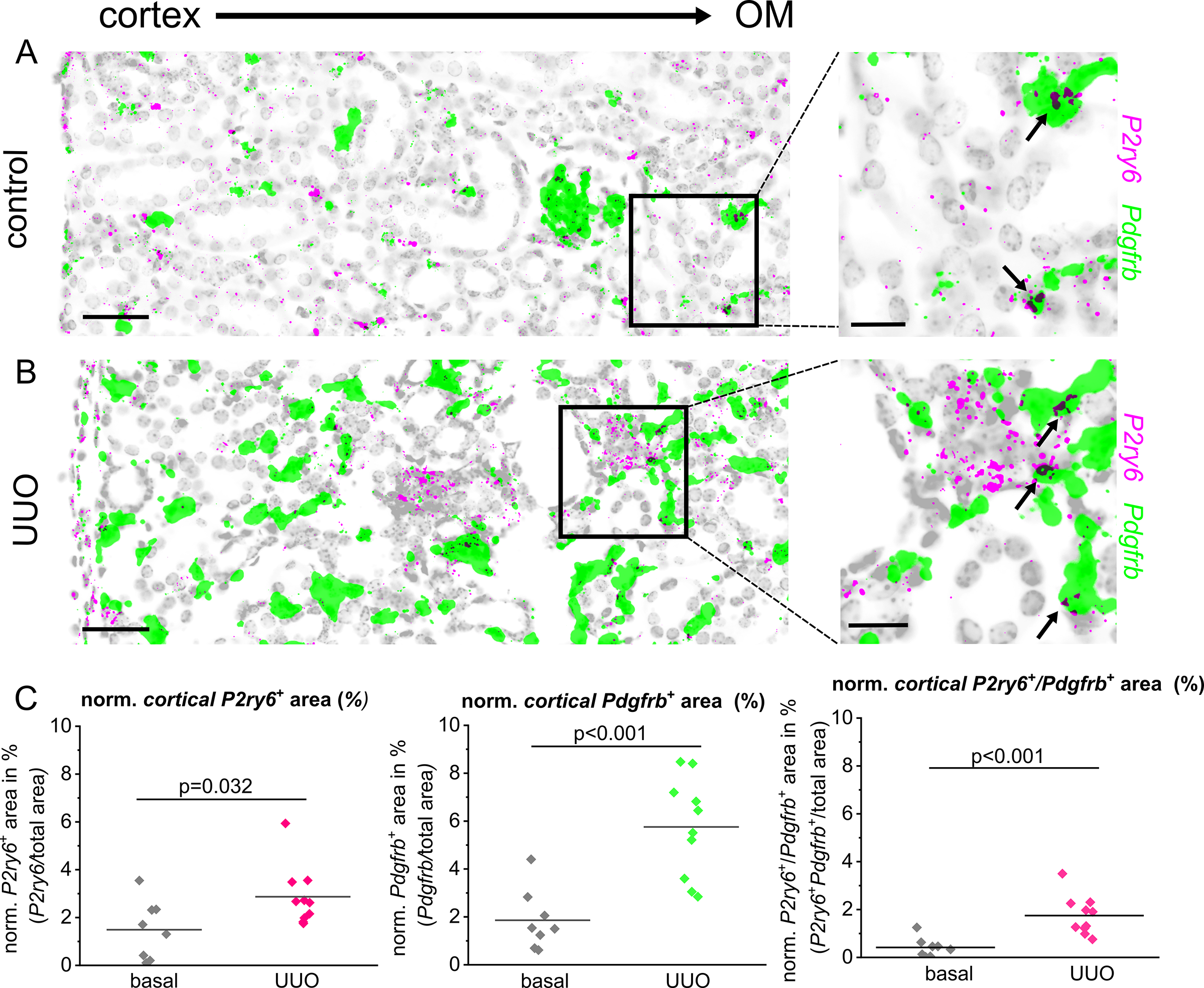

Supplement: Supplementary file 9 — (PNG 1.93 MB) [file 424_2026_3187_Fig10_ESM.png]

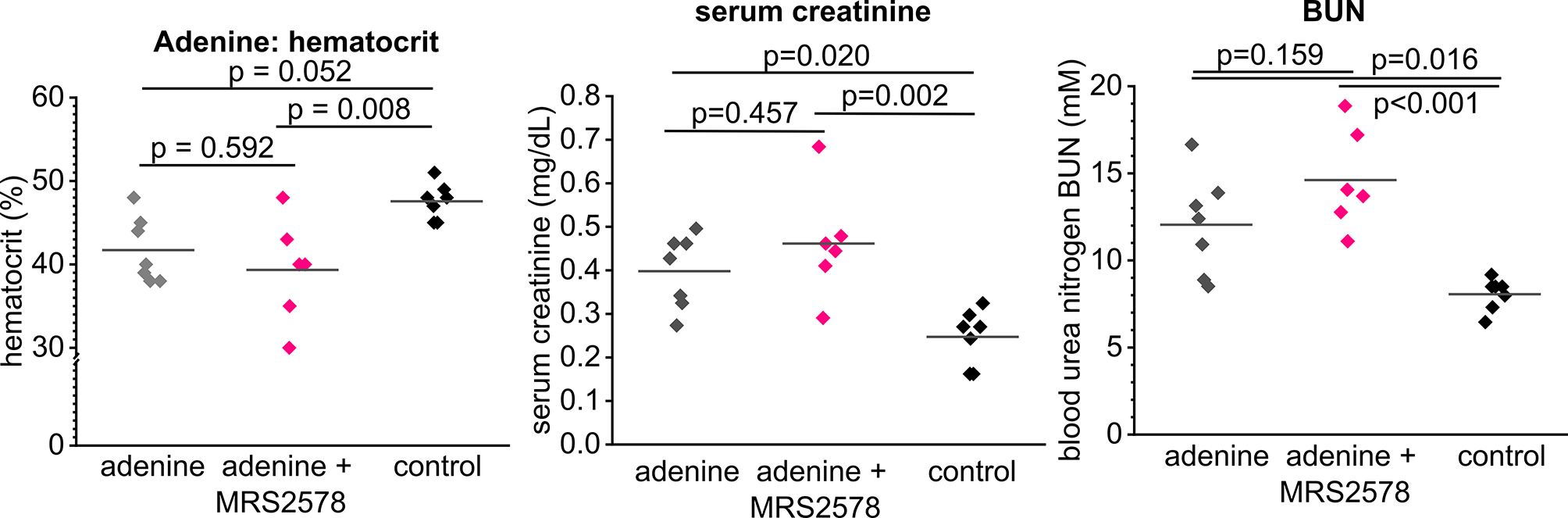

Supplement: Supplementary file 11 — (PNG 141 KB) [file 424_2026_3187_Fig11_ESM.png]

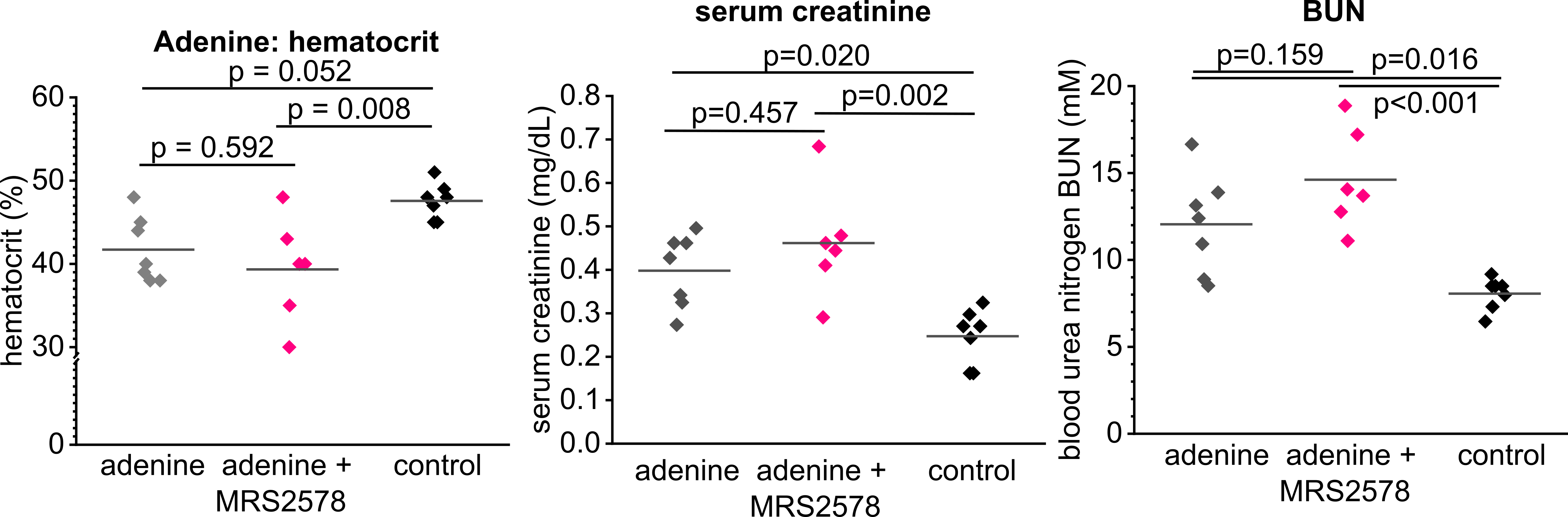

Supplement: Supplementary file 12 — Supplementary Material 6 [file 424_2026_3187_MOESM6_ESM.tiff]

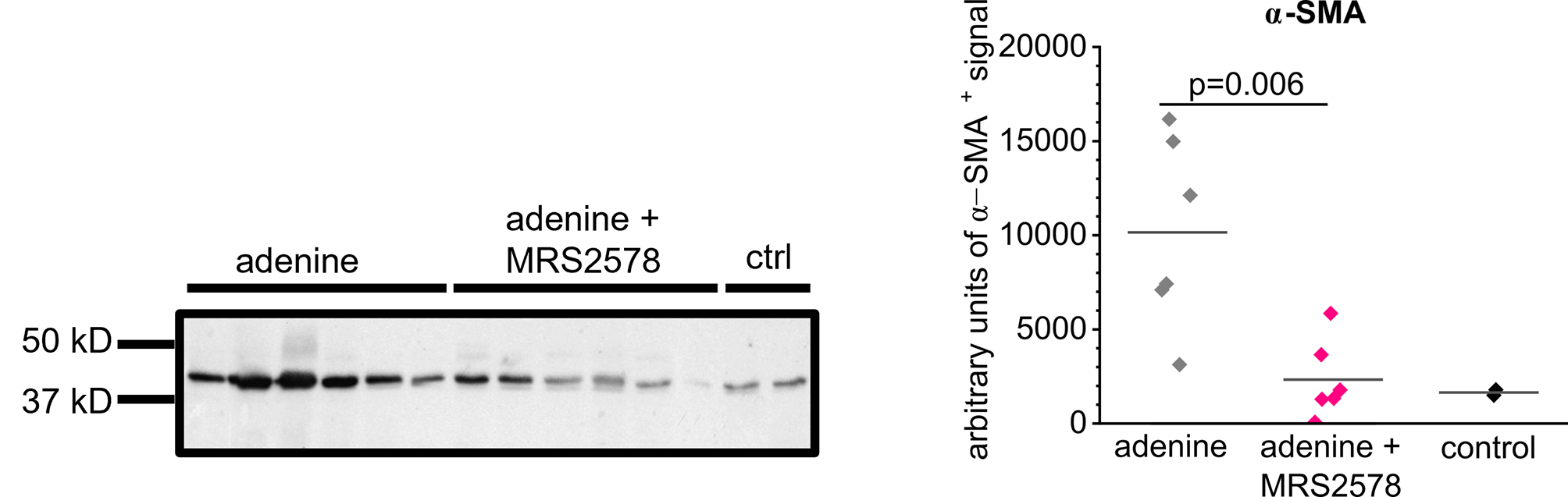

Supplement: Supplementary file 13 — (PNG 174 KB) [file 424_2026_3187_Fig12_ESM.png]

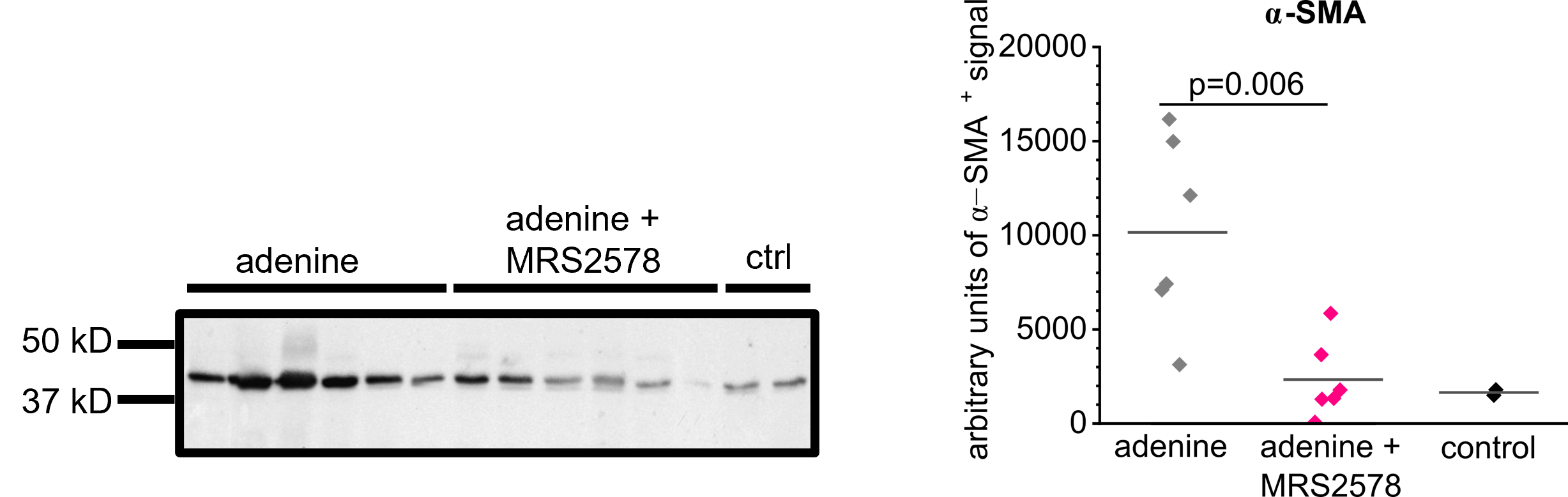

Supplement: Supplementary file 14 — Supplementary Material 7 [file 424_2026_3187_MOESM7_ESM.tif]

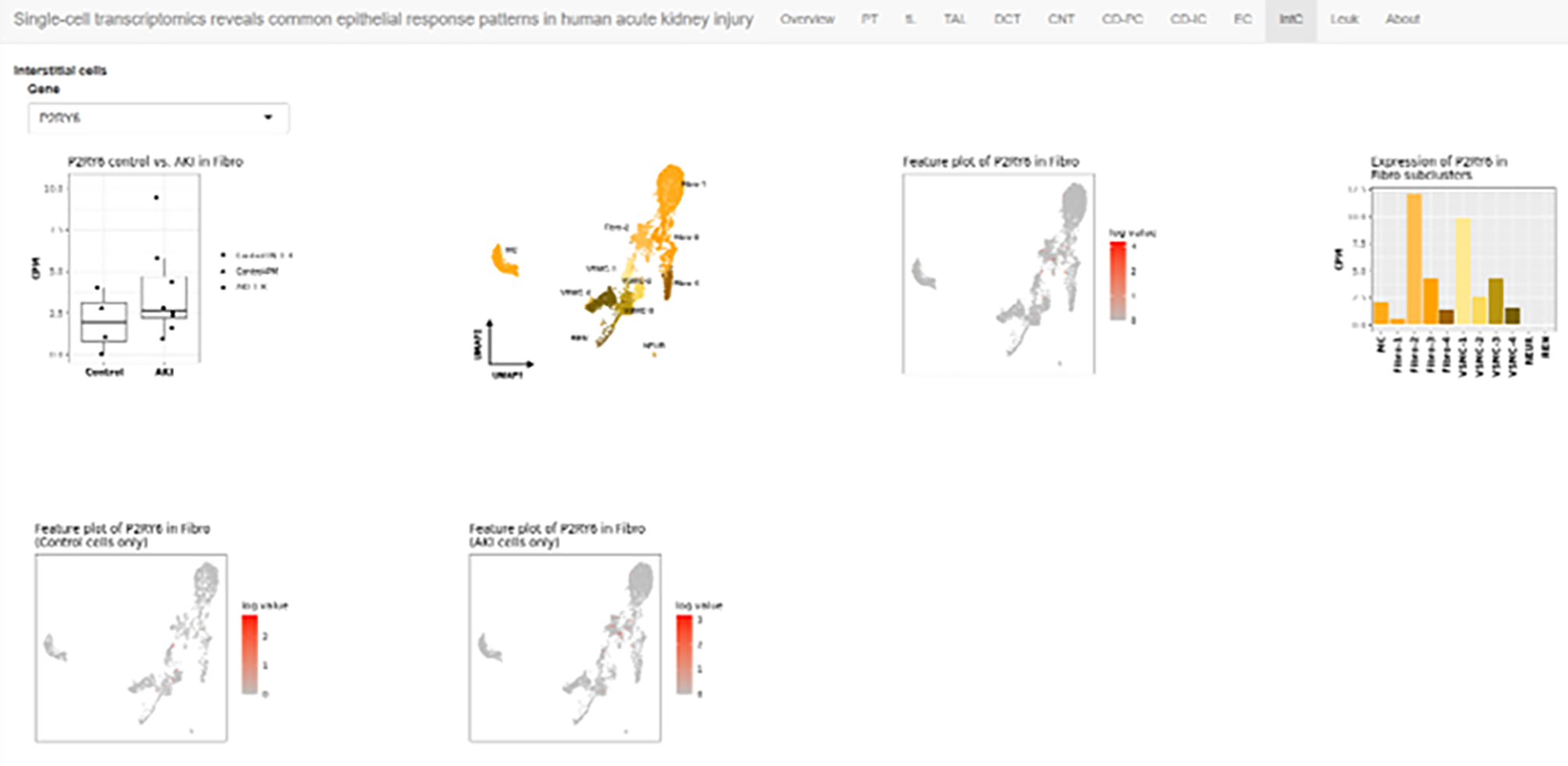

Supplement: Supplementary file 15 — (PNG 267 KB) [file 424_2026_3187_Fig13_ESM.png]

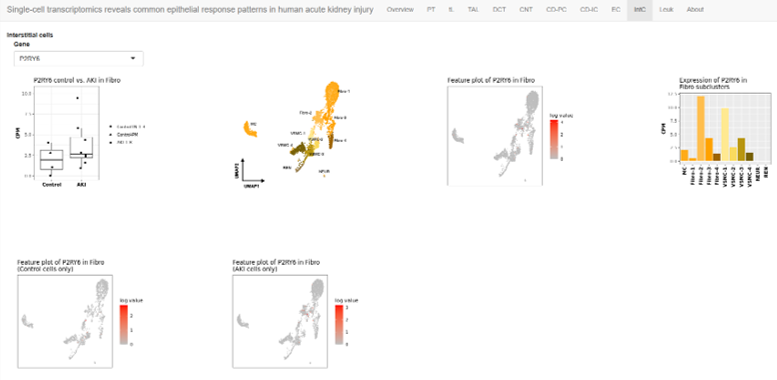

Supplement: Supplementary file 16 — Supplementary Material 8 [file 424_2026_3187_MOESM8_ESM.tif]
